# Antioxidant and Cytoprotective Properties of Polyphenol-Rich Extracts from *Antirhea borbonica* and *Doratoxylon apetalum* against Atherogenic Lipids in Human Endothelial Cells

**DOI:** 10.3390/antiox11010034

**Published:** 2021-12-24

**Authors:** Jonathan Bonneville, Philippe Rondeau, Bryan Veeren, Julien Faccini, Marie-Paule Gonthier, Olivier Meilhac, Cécile Vindis

**Affiliations:** 1 Clinical Investigation Center (CIC) 1436, INSERM 1048, 31400 Toulouse, France; jon.bonneville@gmail.com (J.B.); julien.faccini@free.fr (J.F.); 2 Université de La Réunion, INSERM, UMR 1188 Diabete athérothrombose Réunion Océan Indien (DéTROI), 97400 La Réunion, France; rophil@univ-reunion.fr (P.R.); bryan.veeren@univ-reunion.fr (B.V.); marie-paule.gonthier@univ-reunion.fr (M.-P.G.); 3 Université de Toulouse III Paul Sabatier, 31400 Toulouse, France; 4 CHU de La Réunion, 97448 Saint-Pierre, La Réunion, France

**Keywords:** *Antirhea borbonica*, *Doratoxylon apetalum*, polyphenol, oxidized LDL, vascular cell

## Abstract

The endothelial integrity is the cornerstone of the atherogenic process. Low-density lipoprotein (LDL) oxidation occurring within atheromatous plaques leads to deleterious vascular effects including endothelial cell cytotoxicity. The aim of this study was to evaluate the vascular antioxidant and cytoprotective effects of polyphenol-rich extracts from two medicinal plants from the Reunion Island: *Antirhea borbonica (A. borbonica)*, *Doratoxylon apetalum (D. apetalum)*. The polyphenol-rich extracts were obtained after dissolving each dry plant powder in an aqueous acetonic solution. Quantification of polyphenol content was achieved by the Folin–Ciocalteu assay and total phenol content was expressed as g gallic acid equivalent/100 g plant powder (GAE). Human vascular endothelial cells were incubated with increasing concentrations of polyphenols (1–50 µM GAE) before stimulation with oxidized low-density lipoproteins (oxLDLs). LDL oxidation was assessed by quantification of hydroperoxides and thiobarbituric acid reactive substances (TBARS). Intracellular oxidative stress and antioxidant activity (catalase and superoxide dismutase) were measured after stimulation with oxLDLs. Cell viability and apoptosis were quantified using different assays (MTT, Annexin V staining, cytochrome C release, caspase 3 activation and TUNEL test). *A. borbonica* and *D. apetalum* displayed high levels of polyphenols and limited LDL oxidation as well as oxLDL-induced intracellular oxidative stress in endothelial cells. Polyphenol extracts of *A. borbonica* and *D. apetalum* exerted a protective effect against oxLDL-induced cell apoptosis in a dose-dependent manner (10, 25, and 50 µM GAE) similar to that observed for curcumin, used as positive control. All together, these results showed significant antioxidant and antiapoptotic properties for two plants of the Reunion Island pharmacopeia, *A. borbonica* and *D. apetalum,* suggesting their therapeutic potential to prevent cardiovascular diseases by limiting LDL oxidation and protecting the endothelium.

## 1. Introduction

Among the different risk factors of cardiovascular disease, high levels of low-density lipoprotein cholesterol (LDL-C) remain the major determinant of lipid accumulation within the arterial wall and subsequent atherosclerotic plaque formation. LDL oxidation is responsible for their unregulated uptake by phagocytes, thereby forming foam cells [1]. In the subendothelial space of large arteries, LDL undergo oxidation via different mechanisms involving reactive oxygen species produced by vascular cells and leukocytes in the presence of transition metals such as iron contained in hemin [2]. In the first steps of atherogenesis, oxidized LDLs (oxLDLs) induce the expression of adhesion molecules such as VCAM-1 and ICAM-1 (respectively vascular and intercellular cell adhesion molecules) that promote leukocyte infiltration [3]. Lipids contained in oxLDLs stimulate the expression of pro-angiogenic factors such as vascular endothelial growth factor A by smooth muscle cells, which promotes angiogenesis even in the early stages of atherogenesis [4]. Neoangiogenesis favors intraplaque growth and subsequent hemorrhage that participate in further cholesterol accumulation and erythrocyte extravasation. OxLDLs also display deleterious cytotoxic effects for leukocytes and for different vascular cells, including endothelial cells [5,6]. Disruption of the endothelial layer leads to increased leukocyte extravasation and represents a key factor in destabilizing the plaque towards rupture and ultimately clinical complications.

Different strategies may be used to limit both LDL oxidation and their deleterious effects [7]. Although antioxidant-based dietary interventions may exert a protective effect on cardiovascular disease, supplementation by specific antioxidants such as vitamins E and C, was disappointing [8]. Nutritional approaches that rely on the use of medicinal plants may help to reduce oxidative stress, particularly by counteracting lipid oxidation. Polyphenols are the most abundant antioxidants contained in plant-based beverages [9]. For example, curcumin was long known to inhibit LDL oxidation and the proatherogenic effects of oxLDLs both in vitro and in vivo [10].

*Antirhea borbonica* (*A. borbonica*) and *Doratoxylon apetalum* (*D. apetalum*) are two medicinal plants from Reunion Island traditionally used for their astringency, hemostatic or anti-inflammatory properties. *A. borbonica* is referenced in the French Pharmacopeia, among 27 other medicinal plants from Reunion Island. The choice of these 2 plants relies on their high content in total polyphenols such as phenolic acids and flavonoids, in particular for *D. apetalum* [11]. We showed that the polyphenols contained in these plants have anti-inflammatory and antioxidant effects on adipose cells exposed to different types of bacterial lipopolysaccharides (LPS) [11,12].

In this study, we hypothesized that polyphenol-rich extracts from *A. borbonica* and *D. apetalum* may reduce LDL oxidation and oxidative stress-induced cytotoxicity of oxLDLs on human vascular endothelial cells.

## 2. Materials and Methods

### 2.1. Reagents

Curcumin, 2-thiobarbituric acid (TBA), malondialdehyde (MDA) and 4′,6′-diamino-2-phenylidone (DAPI) were from Sigma-Aldrich (S^t^ Louis, USA). 3-(4,5-dimethylthiazol-2-yl)-2,5-diphenyltetrazolium bromide (MTT) were from Euromedex (Souffelweyersheim, France). SYTO-13, propidium iodide (PI), 6-carboxy-2’,7’-dichlorodihydrofluorescein diacetate (H_2_DCFDA) were from Molecular Probes (Invitrogen, San Diego, CA, USA). Cleaved Caspase-3 antibody was from Abcam (Paris, France) and secondary antibody was from Life technologies (Thermo Fisher Scientific, Carlsbad, CA, USA).

### 2.2. Extraction and Quantification of Polyphenols in Medicinal Plant Extracts

*A. borbonica* and *D. apetalum* medicinal plants were collected in Réunion Island and botanically identified with voucher numbers (Table 1). After airflow drying (45 °C), plant organs were reduced to powder. Polyphenol-rich extracts from medicinal plants were obtained after dissolving each plant powder (2 g) in 20 mL of an aqueous acetonic solution (70%, *v*/*v*) as described previously [11]. A calibration curve was prepared using a standard solution of gallic acid (Sigma–Aldrich, Taufkirchen, Germany). The total phenol content was expressed as mg gallic acid equivalent (GAE)/g plant powder.

### 2.3. Polyphenol Compound Identification by UPLC-UV-ESI-MS/MS

Identification of polyphenols of *A. borbonica* and *D. apetalum* plant extracts was carried out by ultra-high-performance liquid chromatography (UHPLC) coupled with diode array detection and a HESI-Orbitrap mass spectrometer (Q Exactive Plus, Thermo Fisher), according to the method previously used by Delveaux et al., with slight modifications [13].

### 2.4. LDL Isolation and Oxidation

Plasma was obtained from healthy volunteers within the framework of an agreement between INSERM and the French Blood Establishment, in accordance with the provisions of Article L.1243-3 of the Public Health Code, which provides for its use for research purposes. LDL were isolated by ultracentrifugation from a pool of human plasma and mildly oxidized by UV-C in the presence of 5 µM CuSO_4_ as we previously reported [14]. Oxidized LDLs contained 4.2 to 7.4 nmoles of thiobarbituric acid-reactive substances (TBARS)/µg apoB measured according to Yagi et al. [15]. Relative electrophoretic mobility (REM) and 2,4,6-trinitrobenzenesulfonic acid (TNBS) reactive amino groups were 1.2–1.3 times and 85–92% of native LDL, respectively.

### 2.5. Lipid Hydroperoxide Assay

Conjugated diene at 234 nm could not be quantified due to interference of the plant extracts at this wavelength. Lipid hydroperoxides were measured by a modified version of the Ferrous ion Oxidation of Xylenol orange (FOX) assay [16,17]. Briefly, 200 µg apoB/mL of LDL were incubated for 2 h at 37 °C in 500 μL PBS containing 4 μM CuSO_4_ with or without polyphenol-rich extracts. 50 μL of this solution were then added to 450 μL of a solution containing 1 part of solution A (250 mM of sulfuric acid, 2.5 mM of ferrous sulfate and 1 mM xylenol orange) to 9 parts of solution B (4.4 mM of butylated hydroxytoluene in 100% methanol). After 30 min incubation at room temperature (RT) protected from light, the samples were vortex-mixed and then centrifuged for 10 min at 12,000× *g*. The optical density of the supernatant was then measured at 560 nm.

### 2.6. Cell Culture

Human microvascular endothelial cells (HMEC-1) were from ATTC. HMEC-1 cell line is an immortalized human dermal microvascular endothelial cell line expressing von Willebrand’s factor (vWF), cell adhesion molecules such as ICAM-1, and are capable of oxidized LDLs uptake. Cells were grown in MCDB-131 culture medium as we previously described [18]. The cells were starved in serum-free medium 24 h before the experiments.

### 2.7. Thiobarbituric Acid-Reactive Substances Assay

TBARS levels were assessed by the thiobarbituric acid reaction as previously described [15]. TBARS levels were detected in cell culture medium after 6 h of cell exposure to 50 µg apoB/mL native LDL and 1.5 μM CuSO_4_, in the presence or not of various concentrations of polyphenol-rich extracts. Briefly, 100 µL of cell culture medium were added to 100 µL of a 0.375% TBA/15% trichloroacetic acid in 0.25 N HCl, incubated for 10 min at 95 °C, and clarified by centrifugation (400 g for 10 min). The fluorescence was quantified in the supernatant using a TECAN fluorescent spectrophotometer (excitation at 515 nm, emission at 548 nm) by comparison with a standard curve of MDA. TBARS levels were expressed as µM of MDA.

### 2.8. Quantification of Intracellular Reactive Oxygen Species

The generation of intracellular reactive oxygen species (ROS) in endothelial cells was performed as previously described [19] using the 6-carboxy-2’,7’-dichlorodihydrofluorescein diacetate (H_2_DCFDA) ROS-sensitive fluorescent probe (5 µM). Briefly, cells grown in 12-well plates until confluence were incubated with the probe for 30 min at 37 °C, and then stimulated with oxLDLs in the presence or not of each plant extract for 1 h before analysis. The data are expressed as ratio of fluorescence/fluorescence of the nonstimulated control.

### 2.9. Evaluation of Cytotoxicity, Necrosis and Apoptosis

#### 2.9.1. Cell Viability Test

Cytotoxicity was evaluated using the MTT [3-(4,5-dimethylthiazol-2-yl)-2,5-diphenyltetrazolium bromide] test, as we previously described [14]. After cell treatment, MTT (5 mg/mL) were added to each well at a final concentration of 0.5 mg/mL, followed by 4 h of incubation at 37 °C. A solution of dimethylsulfoxide was added to each well to dissolve formazan crystals and absorbance was read at 595 nm. Results are expressed as the percentage of untreated cells.

#### 2.9.2. Apoptotic Versus Necrotic Cell Count by Fluorescence Microscopy

Apoptotic and necrotic cells were counted after staining by two fluorescent dyes, the permeant DNA intercalating green probe SYTO-13 (0.6 µM) and the nonpermeant DNA intercalating red probe propidium iodide (15 µM) using an inverted fluorescence microscope (Fluovert FU, Leitz) [14]. Normal nuclei exhibit a loose, green-colored chromatin whereas primary necrotic cells exhibit loose red-colored chromatin. Apoptotic nuclei showed a condensed yellow/green-colored chromatin associated with nucleus fragmentation, and postapoptotic necrotic cells presented the same morphological features but were red-colored.

#### 2.9.3. Determination of Phosphatidylserine Exposure by Flow Cytometry

Annexin-V-FITC labeling were performed to evaluate phosphatidylserine externalization, an early event of apoptosis. Annexin V staining of HMEC-1 cells was evaluated by flow cytometry using a CytoFLEX (Beckman Coulter) and CytExpert software. Cells were detached with trypsin, washed with PBS and then labeled with 2 μg/mL Annexin V-FITC (dilution in binding buffer, BioLegend) for 15 min at RT. Targeted cell population selected by gating was identified by its typical location in a FSC vs. SSC graph.

#### 2.9.4. In Situ Detection of Cytochrome C

HMEC-1 cells grown on glass coverslips were fixed in PBS-4% paraformaldehyde (PFA) for 10 min at RT, washed and permeabilized with 0.1% TritonX100 for 10 min at RT. After blocking of the non-specific sites with PBS containing 3% bovine serum albumin (BSA), cells were incubated with an anti-cytochrome C primary antibody (1:100 dilution, mouse mAb,12963, Cell Signaling Technology, Saint Quentin Yvelines, France) for 1 h at RT and revealed with Alexa Fluor-conjugated secondary antibody (1:1000 dilution). Cell nuclei were stained with 4′,6-diamidino-2-phenylindole (DAPI) and images were acquired on a Zeiss LSM 780 confocal microscope.

#### 2.9.5. In Situ Detection of Caspase 3 Activation

HMEC-1 cells grown on glass coverslips were fixed in PBS-4% PFA for 10 min at RT. After blocking of the nonspecific sites with PBS containing 2% BSA for 30 min, cells were incubated with a polyclonal anticleaved caspase-3 antibody (1:100 dilution, Abcam, ab13847) for 1 h at RT and revealed with Alexa Fluor-conjugated secondary antibody (1:200 dilution). Cell nuclei were stained with DAPI and images were captured with an Eclipse 80i Nikon fluorescence microscope equipped with a Hamamatsu ORCA-ER digital camera (Life Sciences, Japan). For quantification, 3 representative fields were used for each condition and the fluorescence intensity of images was analyzed with ImageJ software (http://rsb.info.nih.gov/ij/ version 1.32j, accessed on 23 November 2021).

#### 2.9.6. In Situ Cell Apoptosis Detection by TUNEL

Terminal transferase dUTP nick-end (TUNEL) technique was used for the detection of fragmented DNA (in situ Cell Death Detection Kit- Roche). HMEC-1 cells were fixed in PBS-4% PFA for 10 min at RT and processed following the manufacturer’s protocol. Cell nuclei were counterstained with DAPI and images were captured with an Eclipse 80i Nikon fluorescence microscope equipped with a Hamamatsu ORCA-ER digital camera (Life Sciences, Japan). 3 representative fields were used for each condition for quantification of cell death. Results are expressed as a percentage of TUNEL-positive nuclei compared to the total number all nuclei (DAPI-labeled nuclei).

### 2.10. Determination of LDL Cellular Uptake

LDL uptake was measured by using native LDL labeled with the fluorescent lipid dye 3,3′-dioctadecyl-indocarbocyanine (DiI, 300 µg, Molecular Probes), as we previously described [20]. The DiI content was measured by a TECAN fluorescent spectrophotometer (excitation 520 nm, emission 568 nm). Values are expressed as fold of control after normalization to total protein content.

### 2.11. Cell Protein Extraction and Quantification

After treatment, total proteins were obtained from cell lysis by the freeze-thaw method consisting in 5 successive cycles of fast freezing in liquid nitrogen and subsequent thawing in 37 °C water bath. Protein concentration was quantified by the bicinchoninic acid assay (BCA, Sigma).

### 2.12. Catalase and Superoxide Dismutase Activity Assays

The catalase activity assay was carried out on 30 μg of protein lysate. Blanks were measured at 240 nm just before adding 80 μL of H_2_O_2_ (10 mM final) to start the reaction. H_2_O_2_ reduction was monitored by measuring the absorbance every 5 s at 240 nm for 1 min. Catalase activity was calculated using a calibration standard curve with increasing amounts of catalase between 12.5 and 125 units/mL and expressed as international catalytic units per microgram of proteins.

The total superoxide dismutase (SOD) activity was quantified using the cytochrome C reduction assay. Superoxide radicals generated by the xanthine/xanthine oxidase system reduce the ferrycytochrome C into ferrocytochrome C, which then leads to an increase in absorbance at 560 nm. About 20 µg of cell lysate were combined with the reaction mixture (xanthine oxidase, xanthine (0.5 mM), cytochrome C (0.2 mM), KH_2_PO_4_ (50 mM) and EDTA (2 mM)). The reaction was monitored in a microplate reader at 560 nm for 1 min, the SOD activity was calculated using a calibration standard curve of SOD (0 to 6 units/mg) and expressed as international catalytic units per microgram of proteins.

### 2.13. Western Blot Analysis

For western blot analysis, total proteins were extracted in a solubilization buffer as described [14], resolved by SDS-polyacrylamide gel electrophoresis and transferred onto polyvinylidene fluoride (PVDF) membranes (Immobilon, IPVH 00010, Merck Millipore, Molsheim, France). Membranes were probed with caspase 3 antibody (1:1000, rabbit, 9662, Cell Signaling Technology, Saint Quentin Yvelines, France) and revealed with secondary antibodies coupled to horseradish peroxidase (ECL chemoluminescence kit, Amersham, Pittsburgh, PA, USA) using the Chemidoc Touch system (Bio-Rad, Marnes-la-Coquette, France). Stripped membranes were reprobed with an anti-β-actin antibody to control the equal loading of proteins. Quantification of protein bands was performed using Image Lab software (Image Lab 6.0.1, Bio-Rad, Marnes–la–Coquette, France).

### 2.14. Statistical Analysis

Results were expressed as means ± SEM. Differences between two groups were analyzed by an unpaired two-tailed Student’s *t*-test. Differences between more than two groups were analyzed by one-way analysis of variance (ANOVA) with Tukey’s *posthoc* test for multiple comparisons. Statistical significance was set to *p* < 0.05.

## 3. Results

### 3.1. Polyphenol Content of D. apetalum and A. borbonica Plant Extracts

Dietary polyphenols represent the most abundant antioxidants provided by the human diet. Previous studies performed in our laboratory have led us to focus on 2 plants from Reunion Island widely used by the population for their anti-inflammatory properties, namely *D. apetalum* and *A. borbonica*. These 2 plants contain large amounts of polyphenols and were shown to reduce the production of interleukin-6 and MCP-1 in pre-adipocytes exposed to TNF-a, H_2_O_2_ or lipopolysaccharides [11]. The identification of polyphenols of acetonic extracts of *D. apetalum* and *A. borbonica*, was performed by mass spectroscopy. Figures S1 and S2 show the total ion chromatogram (TIC) and chromatographic profiles (recorded at 315 nm) of *D. apetalum* and *A. borbonica* extracts, respectively. For *D. apetalum,* a total of 17 main compounds, numbered according to their elution order, were identified and reported in Table 2 with their retention time, experimental m/z mass, corresponding chemical formulas and their main MS/MS fragments.

The main polyphenols identified were three kaempferol derivatives (kaempferol 3-*O*-rutinoside, kaempferol 3-*O*-hexoside and kaempferol 3-*O*-(6-malonyl-hexoside)), two quercetin derivatives (quercetin 3-*O*-rutinoside (Rutin) and quercetin 3-*O*-hexoside) and two procyanicin derivatives (dimer type B and trimer type C). Several minority polyphenols were also identified such as apigenin or gallic acid 4-*O*-glucoside. For *A. borbonica,* a total of 21 main compounds were identified and reported in Table 3. The main polyphenols identified were several dicaffeoylquinic acids derivatives, caffeic acid, and quercetin derivatives. Coumaric acid, 5-feruloylquinic acid and protocatechuic acid are three polyphenols identified among the minority compounds.

Total polyphenol content of acetonic extracts obtained from these 2 medicinal plants was also evaluated by using Folin–Ciocalteu assay. As shown in Table 4, *D. apetalum* extract displays a higher polyphenol content (3.79 mg GAE/g plant) compared to *A. borbonica* extract (1.98 mg GAE/g plant). Description and identification of the different polyphenols contained in these plants was previously published by our group [11].

### 3.2. A. borbonica and D. apetalum Extracts Prevent the Oxidation of LDL

**Figure 1:**
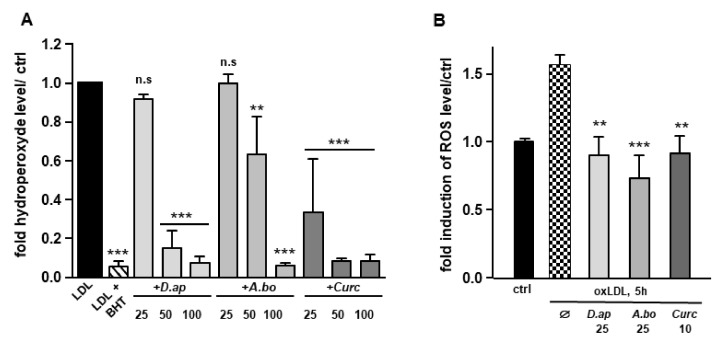
*D. apetalum* and *A. borbonica* extracts exhibit antioxidant properties. Antioxidant properties of various concentrations of polyphenol-rich extracts from *D. apetalum* and *A. borbonica* or curcumin were evaluated on LDL oxidation and oxLDL-induced intracellular ROS production in HMEC-1 cells. (**A**) Fold increase of hydroperoxide formation assayed by xylenol orange test. LDL are incubated for 2 h at 37 °C with 4 µM CuSO_4_ with or without plant extracts. Hydroperoxide formation was followed by colorimetric reaction, using xylenol orange; (**B**) inhibition of oxLDL-induced intracellular ROS production. HMEC-1 cells were pre-treated with plant extracts 2 h before addition of oxLDLs for 5 h. increase in intracellular ROS was quantified using H_2_DCFDA fluorescent probe and expressed as fold of induction of ROS levels compared with that of control (without oxLDLs). Results are expressed as means ± SEM of at least 3 independent experiments. ** *p* < 0.01, *** *p* < 0.001, compared to control (ctrl) (LDL) [A]; ** *p* < 0.01, *** *p* < 0.001, compared to oxLDLs [B] using one-way ANOVA with posthoc Tukey’s test.

Since polyphenols are widely recognized antioxidants, we tested the ability of *D. apetalum* and *A. borbonica* extracts to inhibit copper-induced LDL oxidation by measuring the formation of hydroperoxides. LDL (200 μg apoB/mL) isolated from a pool of plasma from healthy volunteers were incubated with 4 μM CuSO_4_ and the formation of hydroperoxides was monitored for 2 h. Ferric products generated from hydroperoxides were measured as xylenol orange complexes at 560 nm [21]. Butylated hydroxytoluene (BHT) and curcumin (powerful antioxidants) were used as positive controls for the inhibition of LDL oxidation. Results reported in Figure 1A show that *D. apetalum* and *A. borbonica* extracts significantly reduced the formation of copper-induced LDL hydroperoxides at 50 and 100 μM GAE, while curcumin significantly exerted its antioxidant effect at all concentrations tested (25, 50 and 100 μM). *D. apetalum* extract is significantly more efficient at 50 μM GAE than *A. borbonica* extract.

Vascular cells and particularly endothelial cells produce ROS, promoting LDL oxidation [22]. We tested the capacity of plant extracts to limit cell-induced LDL oxidation. Both polyphenol-rich extracts (10–50 μM GAE) and curcumin (5–10 μM) were able to significantly prevent TBARS formation in cell-incubated LDL (50 μg apoB/mL) (Table 5). Altogether, these results demonstrate that *A. borbonica* and *D. apetalum* extracts exhibit a robust free radical-scavenging activity against both copper- and cell-induced LDL oxidation.

Serum- starved HMEC-1 cells were preincubated or not (control, vehicle PBS) for 2 h with plant extracts (10, 25 or 50 µM) or curcumin (1, 5 or 10 µM) before addition of 200 μg apoB/mL of LDL for 6 h. Lipid peroxidation was measured in conditioned medium by TBARS assay and expressed as µM of MDA. All data are expressed as means ± SEM of at least three independent experiments. n.s: nonsignificant.

### 3.3. Oxidative Stress and Antioxidant Response Induced by oxLDLs in Endothelial Cells Are Inhibited by A. borbonica and D. apetalum Extracts

OxLDLs induce an intense oxidative stress in vascular cells [14,23,24]. The capacity of *A. borbonica* and *D. apetalum* extracts to limit oxLDL-induced generation of ROS was evaluated in HMEC-1 cells. OxLDL induced a 1.55-fold increase in ROS production respectively after 5 h of HMEC-1 cells stimulation. Both polyphenol-rich extracts exhibited a significant antioxidant activity similar to that of curcumin, used as a positive control (Figure 1B). Interestingly, after 5 h of treatment of cells with oxidized LDL, *A. borbonica* extract displayed a more significant antioxidant effect than *D. apetalum* extract. We then tested whether the polyphenol extracts interfered with LDL binding and/or uptake using fluorescently labeled lipoproteins (Figure S1, Supplementary Materials). No significant difference in LDL uptake was observed when HMEC-1 cells were coincubated or not with 25 μM GAE of plant extracts, or with 10 μM of curcumin.

**Figure 2:**
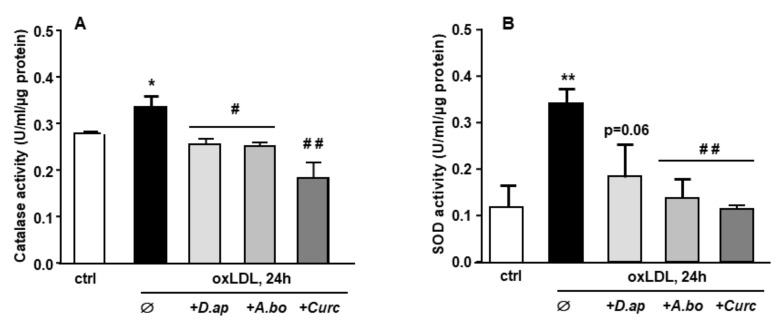
*D. apetalum* and *A. borbonica* extracts prevent oxLDL-induced cellular oxidative stress. Effects of polyphenol-rich extracts from *D. Apetalum* and *A. borbonica* or curcumin on antioxidant activity of oxLDL-stimulated HMEC-1 cells. Subconfluent serum-starved HMEC-1 cells were preincubated for 2 h with *D. apetalum* (25 µM), *A. borbonica* (25 µM) polyphenol-rich extracts or curcumin (10 µM) before addition of oxLDLs (200 μg apoB/mL) for an additional 12 h. (**A**) Catalase and (**B**) total SOD activities were expressed as catalytic units per μg of proteins as means ± SEM of at least 3 independent experiments. * *p* < 0.05; ** *p* < 0.01 compared to control (ctrl, vehicle PBS); # *p* < 0.05; ## *p* < 0.01 compared to oxLDLs were calculated using one-way ANOVA with Tukey’s posthoc test.

When exposed to an oxidative stimulus such as oxLDLs, endothelial cells trigger an antioxidant response by stimulating the production of enzymes in charge of O_2_^−^ and H_2_O_2_ detoxification, namely superoxide dismutase (SOD) and catalase, respectively. As shown in Figure 2, both catalase and SOD activities were increased after HMEC-1 cells incubation with oxLDLs. In the presence of plant extracts, the antioxidant response was no longer observed, suggesting that *A. borbonica* and *D. apetalum* extracts inhibit the oxidative stress generated by oxLDLs, upstream of the induction of SOD and catalase activities.

### 3.4. A. borbonica and D. apetalum Extracts Inhibit the Cytotoxic Effects of oxLDLs on Endothelial Cells

**Figure 3:**
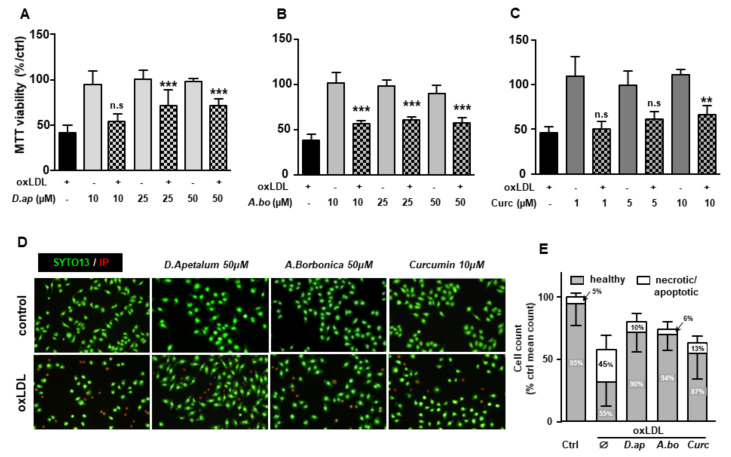
*D. apetalum* and *A. borbonica* extracts exert cytoprotective effects on oxLDL-stimulated HMEC-1 cells. Analysis of cell viability in HMEC-1 cells was evaluated by MTT assay. Subconfluent serum-starved HMEC-1 cells were preincubated for 2 h with (**A**) *D. apetalum* (10–50 µM), (**B**) *A. borbonica* (10–50 µM) and (**C**) curcumin (1–10 µM) before addition of oxLDLs (200 μg apoB/mL), for 24 h. MTT reduction and cell viability was analyzed as described. Results are expressed as percentage of untreated control cells (vehicle, PBS) and represent mean ± SEM of 4 independent experiments. (**D**) SYTO-13/PI staining of HMEC-1 cells pretreated with plant extracts or curcumin followed by addition of oxLDLs (200 μ*g* apoB/mL, for 24 h), images show protective effect of plant extracts and curcumin towards oxLDL-induced apoptosis. (**E**) Living, apoptotic, and necrotic cells were counted after staining by SYTO-13/PI as described. Results are expressed as percentage and represent mean ± SEM of at least 4 independent experiments. ** *p* < 0.01; *** *p* < 0.001 were calculated using one-way ANOVA with Tukey’s post hoc test. n.s: nonsignificant.

OxLDLs are well documented for their cytotoxic effects on endothelial cells [5,25]. We tested the ability of *A. borbonica* and *D. apetalum* extracts to limit apoptosis induced by oxLDLs under our experimental conditions. Different techniques were used to quantify the overall cell viability and specifically apoptosis, including the MTT reduction by the respiratory chain and other electron transport systems, which serve as an estimate for the metabolic activity of living cells, the cell membrane permeability (propidium iodide, PI), morphological nuclear changes (SYTO 13), phosphatidylserine exposure (annexin V membrane binding), cytochrome C release and caspase 3 activation. The MTT test showed that extracts of *A. borbonica* (10–50 μM) and *D. apetalum* (25–50 μM) together with curcumin (10 μM) limited oxLDL-induced mitochondrial dysfunction (Figure 3A–C). This suggests that both plant extracts exhibited a cytoprotective effect capable of counteracting oxLDL toxicity for concentrations as low as 10 µM GAE for *A. borbonica* and 25 µM GAE for *D. apetalum.*

**Figure 4:**
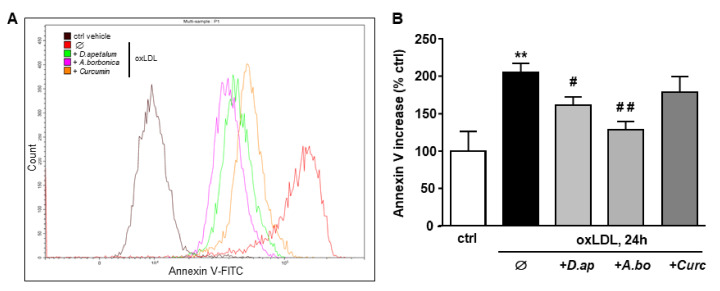
*D. apetalum* and *A. borbonica* extracts prevent oxLDL-induced phosphatidylserine exposure. Subconfluent serum-starved HMEC-1 cells were preincubated for 2 h with *D. apetalum* (25 µM), *A. borbonica* (25 µM) polyphenol-rich extracts or curcumin (10 µM) before addition of oxLDLs (200 μg apoB/mL) for an additional 24 h. (**A**) Illustration of detection of Annexin V binding in HMEC-1 cells quantified by flow cytometry using Annexin V-FITC probe. (**B**) Graph represents percentage of Annexin V fluorescence relative to untreated control HMEC-1 cells (vehicle PBS) in each condition. Results are expressed as means ± SEM of 3 independent experiments. ** *p* < 0.01 vs. control (ctrl); # *p* < 0.05; ## *p* < 0.01 vs. oxLDL using one-way ANOVA with Tukey’s posthoc test.

**Figure 5:**
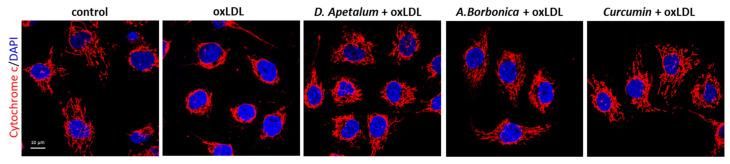
*D. apetalum* and *A. borbonica* extracts inhibit oxLDL-induced cytochrome C release from mitochondria to cytosol. Subconfluent serum-starved HMEC-1 cells were preincubated for 2 h with D. apetalum (25 µM), A. borbonica (25 µM) polyphenol-rich extracts or curcumin (10 µM) before addition of oxLDLs (200 μg apoB/mL) for an additional 24 h, control condition corresponds to untreated HMEC-1 cells (vehicle, PBS). Images are representative of immunofluorescence staining of cytochrome C (red) and cell nuclei (blue) stained with DAPI. Scale bar = 10 µm.

The plant extracts also significantly reduced apoptosis and necrosis as shown by various tests. Using SYTO 13/PI staining, nuclear morphology and membrane integrity could be assessed simultaneously. SYTO 13 is a green, fluorescent nucleic acid dye shown to stain live cells (Figure 3D). OxLDLs induced a nuclear condensation and fragmentation, accompanied by cell shrinkage, and eventually increased membrane permeability. In Figure 3E, the total number of adherent cells was significantly decreased upon incubation with oxLDLs (42% decrease, *p* < 0.001 relative to control cells). Among the remaining adherent cells, apoptosis and postapoptotic necrosis features were observed in oxLDL-treated cells (about 45% of remaining cells) whereas pre-treatment of endothelial cells with the plant extracts significantly limited this proapoptotic effect of oxLDLs (10.0% and 5.8% of remaining cells with *D. apetalum* and *A. borbonica* respectively, (*p* < 0.001 relative to oxLDL), and 12.7% of remaining cells with curcumin (*p* < 0.05 relative to oxLDLs) (Figure 3E). This result was further confirmed by quantifying annexin-V staining, which was significantly limited by pre-incubation with the plant extracts, whereas curcumin only partially reduced the effects of oxLDLs (Figure 4). We previously demonstrated that the mechanism of apoptosis triggered by oxLDLs involved an intrinsic mitochondrial pathway leading to caspase 3 activation [15]. As shown in Figure 5, immunofluorescence staining revealed the localization of cytochrome C in mitochondria in untreated control cells, whereas after oxLDL treatment we observed a perinuclear and cytosolic relocation of the cytochrome C. Importantly, the release of the cytochrome C from mitochondria to cytosol is prevented by the pre-treatment of endothelial cells with the plant extracts and curcumin.

**Figure 6:**
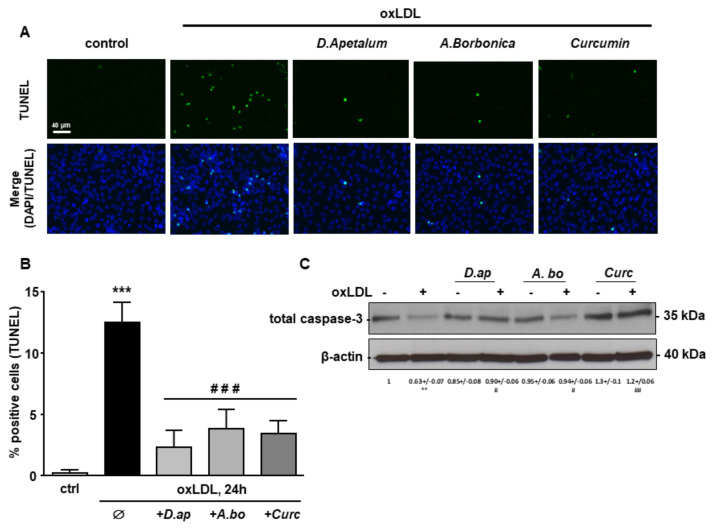
*D. apetalum* and *A. borbonica* extracts inhibit oxLDL-induced caspase 3 activation. Subconfluent serum-starved HMEC-1 cells were preincubated for 2 h with *D. apetalum* (25 µM), *A. borbonica* (25 µM) polyphenol-rich extracts or curcumin (10 µM) before addition of oxLDLs (200 μg apoB/mL) for an additional 24 h. (**A**) Immunofluorescence staining of cleaved caspase-3 expression in HMEC-1 cells. Green signal represents cleaved caspase-3 levels. Cell nuclei are stained with DAPI (blue). Scale bar = 40 µm; (**B**) graph represents means ± SD of cleaved caspase-3 fluorescence intensity. Results are expressed as means ± SEM of three independent experiments. (**C**) Western-blot analysis of total caspase-3 expression in HMEC-1 cells. Densitometric analyses of expression level of caspase-3 protein upon different conditions are indicated. data are expressed as mean ± SEM of four independent experiments. *** *p* < 0.001 vs. control; ### *p* < 0.001 vs. oxLDL using one-way ANOVA with Tukey’s posthoc test.

**Figure 7:**
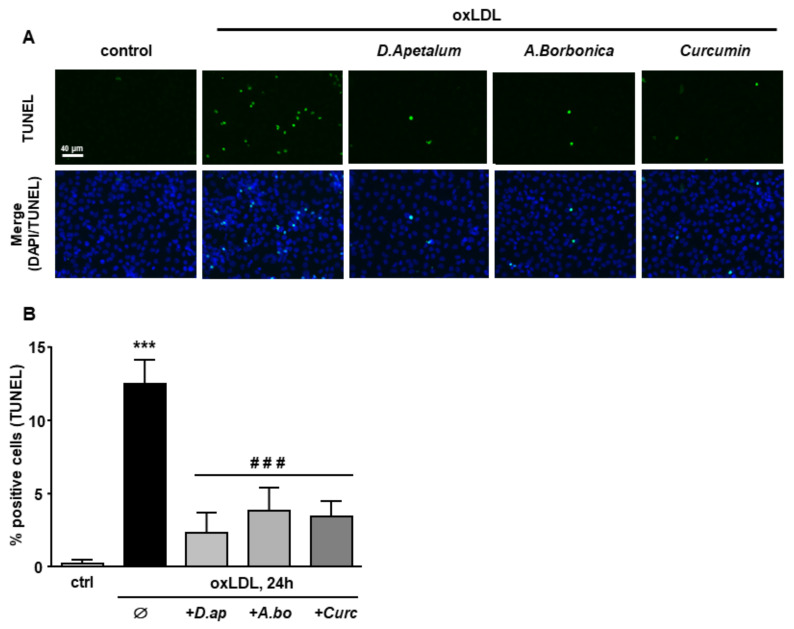
*D. apetalum* and *A. borbonica* extracts limit oxLDL-induced DNA fragmentation. Subconfluent serum-starved HMEC-1 cells were preincubated for 2 h with *D. apetalum* (25 µM), *A. borbonica* (25 µM) polyphenol-rich extracts or curcumin (10 µM) before addition of oxLDLs (200 μg apoB/mL) for an additional 24 h. (**A**) Analysis of DNA fragmentation by TUNEL assay in HMEC-1 cells (green). Cell nuclei are stained with DAPI (blue). Scale bar = 40 µm; (**B**) graph represents percentage of TUNEL-positive cells. Results are expressed as means ± SEM of 3 independent experiments; *** *p* < 0.001 vs. control (ctrl); ### *p* < 0.001 vs. oxLDL using one-way ANOVA with Tukey’s posthoc test.

Finally, caspase 3 activation and DNA fragmentation (determined by the TUNEL method), both characteristics of the late apoptotic process, were increased by incubation with oxLDLs and significantly reduced by pretreatment with *A. borbonica* extract and curcumin (Figure 6 and Figure 7).

Our results demonstrate that polyphenol-rich extracts exerted a potent cytoprotective effect on endothelial cells exposed to oxLDLs, suggesting that they could limit the atherogenic process.

## 4. Discussion

Taken together, our data demonstrate that *A. borbonica* and *D. apetalum* medicinal plant extracts as well as curcumin efficiently inhibit LDL oxidation and could potentially limit intracellular oxidative stress in endothelial cells after exposition to oxLDLs. In addition, the polyphenol-rich extracts of both plants exerted a significant cytoprotective effect as shown by various assays including MTT reduction, SYTO 13/propidium iodide staining, annexin V exposure, caspase 3 activation and TUNEL. For the first time, the antiapoptotic effects against oxLDL-induced toxicity are reported for these plant extracts.

In Reunion Island, medicinal plants are widely used for their anti-inflammatory, antidiabetic, diuretic, or dermatological properties either as herbal tea (infusion or decoction) or in external use (skin application). *A. borbonica* is referenced at the French pharmacopeia for its healing and hemostatic properties, but is widely used as herbal tea. *D. apetalum* is also used against skin irritation (added to the bath). Polyphenol-rich extracts from both plants were shown to contain high amounts of these antioxidants [11]; the antioxidant activity of *A. borbonica* was reported for many years [26]. The present study is the first to evaluate the potential of these medicinal plants from Reunion Island to limit LDL oxidation and subsequent cytotoxic effects on endothelial cells. The polyphenol composition of *D. apetalum* and *A. borbonica* was previously published by our laboratory [11]. Both plant extracts have in common kaempferol-*O*-hexoside-*O*-rhamnoside but have different compositions regarding other major polyphenols. Indeed, *A. borbonica* contains mainly caffeic acid and dicaffeoylquinic acids whereas *D. apetalum* contains procyanidins including dimer type A/B and trimer type A, coumaric acid, epicatechin, quercetin-*O*-rutinoside, kaempferol 3-*O*-hexoside. Most of these compounds were previously reported to inhibit LDL oxidation. For example, chlorogenic acid reacts with peroxyl radicals to limit LDL oxidation [27], and can be incorporated into LDL after drinking polyphenol-containing beverages such as coffee, thereby increasing the resistance of LDL to oxidation [28]. Chlorogenic acid was also reported to protect against atherosclerosis in ApoE KO mice [29]. Procyanidin moieties were shown to limit LDL oxidation, regardless of their molecular weight for monomers, dimers, and trimers whereas hexamers are less effective [30]. Other polyphenols such as flavonoids including epicatechin, quercetin and kaempferol also exhibit radical-scavenging effects and are important inhibitors of LDL oxidation [31]. Curcumin was shown to inhibit LDL oxidation both in vitro [10,32] and in vivo, after oral administration in a rabbit model of atherosclerosis [33].

Even if in vivo polyphenol bioavailability may depend on the interindividual variability and may differ from one polyphenol to another, circulating polyphenol concentrations reach the range of μM. The doses selected in the present study were 25, 50, and 100 μM for plant polyphenols, and 1, 5 and 10 µM for curcumin. Although these doses are higher than the physiological concentrations reported in nutritional interventions, such pharmacological concentrations were broadly used in similar experiments in the literature [18,34] as well as in our published studies using *D. apetalum* and *A. borbonica* polyphenols [11,12] or pure polyphenols like curcumin [35,36]. Higher curcumin concentrations (12.5–100 µM) were reported to induce cell death in endothelial cells [37].

Specific polyphenols such as epicatechin and its derivatives procyanidins were identified in *D. apetalum* while specific caffeic acid esters were detected in *A. borbonica*. In parallel, both plant extracts exhibited similar polyphenols such as kaempferol or quercetin recognized as a flavonoid exerting a strong antioxidant effect due to its B-ring hydroxylation that facilitates electron delocalization on the aromatic ring [38]. Here, the similar effects induced by both plant extracts may result from the presence of similar potent antioxidant polyphenols such as quercetin. Moreover, we used similar doses of total polyphenols from both plant extracts. This may contribute to a similar antioxidant capacity of both extracts as we previously demonstrated through DPPH radical-scavenging activity assay for *D. apetalum* and *A. borbonica* [11]. Concerning the antioxidant potential of a polyphenol, it may depend on the structure of the polyphenol considered, but also on the experimental method used. In the study by Hatia et al. [35], we compared by different in vitro assays the antioxidant capacity of 26 polyphenols including epicatechin, caffeic acid, or quercetin found in the two plant extracts. We found that all polyphenols tested at the same dose exerted antioxidant effects, but the magnitude of the antioxidant capacity depended on the method used. For instance, epicatechin and quercetin exerted a similar reducing activity via Folin–Ciocalteu assay. Epicatechin and caffeic acid exhibited a similar radical-scavenging property with DPPH assay. Quercetin and caffeic acid have a close radical-scavenging capacity, as assessed by the Trolox test. Thus, despite the difference in polyphenolic composition of the plant extracts, the use of similar doses of total polyphenols may contribute to explain the similar effects of plant extracts.

We previously demonstrated that oxLDLs are capable of inducing cytotoxic effects in a variety of cell type including endothelial cells [5,25]. These cytotoxic effects have also been shown to be inhibited by various antioxidant strategies such as polyphenols supplementation [39]. Other studies have shown that polyphenol-rich plant extracts, such as those from *Hibiscus sabdariffa* leaf, can inhibit oxLDL-induced endothelial cell apoptosis via an upregulation of the autophagic pathway. In particular, epicatechin gallate contained in this extract was found to be responsible for this protective effect [40].

In bovine aortic endothelial cells, oxLDLs were able to induce a significant intracellular oxidative stress induced as early as 5 min after stimulation, reaching a plateau after 30–45 min, via an interaction with LOX-1 (Lectin-like oxLDL receptor) [23]. Similarly, Yen et al. reported H_2_O_2_ generation measured after 30 min of oxLDL incubation with HUVECs. OxLDLs used in the present study may utilize, at least in part, the classical internalization pathway via the LDL receptor since oxidative modifications of proteins and lipids are not sufficient to fully direct these lipoproteins towards scavenger receptors [41]. After binding to their receptors, oxLDLs are internalized into endosomes and then lysosomes in which oxidatively modified products can diffuse into the cytosol. Intracellular ROS formation from peroxisomes, NADPH oxidase, mitochondrial respiratory chain and lipoxygenases is then observed. Various signaling pathways are then activated, leading to an intracellular calcium peak, mitochondrial intrinsic pathway activation and subsequent changes in the expression of pro- and anti-apoptotic genes [14,42,43]. Other genes involved in the antioxidant defense can also be induced [7]. Lipid peroxides such HPODE and HODE (13-hydroperoxyoctadecadienoic and 13-hydroxyoctadecadienoic acids, respectively) that can be found in oxLDLs and were reported to induce the expression of catalase [44].

In the present study, we report an increase in intracellular H_2_O_2_ formation assessed by H_2_DCFHDA fluorescence at 1 and 5 h after stimulation with oxLDLs. It is suggested that the intracellular oxidative stress induced by oxLDLs and inhibited by both plant extracts and curcumin may be upstream of the cytotoxic cascade. Literature data report that antioxidant effects of polyphenols may result from different mechanisms involving the direct neutralization of ROS, or indirectly through the modulation of ROS-producing and ROS-detoxifying enzymes [45]. Firstly, due to their phenolic structure, polyphenols are able to directly scavenge and reduce free radicals. In the case of polyphenols from *D. apetalum* and *A. borbonica*, such radical-scavenging and reducing capacities were already demonstrated in our previous studies [11,12]. Secondly, polyphenols from *D. apetalum* and *A. borbonica* may exert antioxidant properties through their ability to modulate the production of redox enzymes [45]. In endothelial cell models, we previously showed that *A. borbonica* polyphenols improved intracellular ROS levels, the expression of genes encoding NOX4, GPx or HO1 as well as SOD antioxidant activity deregulated by oxidative stress conditions [46,47]. *D. apetalum* polyphenols were also found to improve mitochondrial molecular targets involved in redox status in endothelial cell model during oxidative stress conditions [48]. Both the transcriptional factors Nrf2 and NFĸB play opposite pivotal roles in the regulation of the production of redox enzymes. In endothelial cell model exposed to oxidative stress conditions, we reported that *A. borbonica* polyphenols helped to preserve the antioxidant defense system by reducing the nuclear translocation of Nrf2 and elevating Nfr2 gene expression [46]. In parallel, *A. borbonica* polyphenols counteracted the elevation of NFĸB activity and gene expression mediated by oxidative stress [47,49]. Altogether, these findings provide evidence for the several possible mechanisms of action of antioxidant polyphenols from *D. apetalum* and *A. borbonica* plants. In the present study, the ability of plant polyphenols to improve redox targets such as catalase and SOD activities or intracellular ROS levels deregulated by ox-LDL, may involve these several possible mechanisms of action.

The bioavailability of polyphenols and their cellular distribution are not clearly demonstrated, in particular whether they accumulate within endothelial cells and via which potential receptor [50]. Using a cerebral endothelial cell line (bEnd.3), we recently demonstrated that caffeic acid, quercetin and its methylated metabolite isorhamnetin could accumulate intracellularly whereas chlorogenic and gallic acids were only able to bind the plasma membrane [49]. Quercitrin, a glycoside formed from quercetin and rhamnose, showed potential to block oxLDL uptake, suggesting that some polyphenol could act at the extracellular level [51]. Similarly, procyanidins were shown to be potent inhibitors of LOX-1/oxLDL interactions [52]. In our study, the uptake of LDLs is not modified in the presence of plant extracts, suggesting that the protective effects are not mediated by potential interference between polyphenols and LDL receptors during coincubation.

Some recent studies suggest that polyphenols can be transported across the blood-brain barrier and exert their protective effects via the mTOR signaling pathway [53]. Resveratrol was shown to be taken up by endothelial cells via both passive and active transport involving SGLT-1 (sodium-dependent glucose transporter 1) [54]. In our study, the mechanism of action of the plant extracts remains to be elucidated and it is not excluded that the different polyphenols act at both the extracellular and intracellular levels.

Endothelial dysfunction is a hallmark of atherogenesis that is involved in all stages from initial lipid accumulation to plaque rupture [55]. Mildly oxLDLs were shown to induce massive apoptosis of endothelial cells via activation of various intracellular pro-apoptotic signaling pathways, ultimately leading to DNA fragmentation and cell detachment [25]. Here, we demonstrated that plant extracts were able to prevent oxLDL-induced endothelial cell apoptosis using different assays to evaluate apoptosis (annexin V exposure, cytochrome C release caspase 3 activation, TUNEL, nuclear morphology, membrane permeability). Several studies reported the endothelial protective effects of different polyphenols against oxLDL cytotoxicity. Resveratrol cytoprotection is particularly documented [56,57,58] but other polyphenols were demonstrated to limit the deleterious effects of oxLDLs [40,59]. The intracellular signaling pathway involved in polyphenol protection may involve PI3K/Akt/eNOS [59]. In our study, we did not investigate the potential intracellular pathway that might be the target of polyphenols. However, it is likely that plant extracts exert their protective effect at different levels. In addition to the expected antioxidant effect, polyphenols might act after the initiation of intracellular oxidative stress because they remained cytoprotective even when added up to 12 h after oxLDLs (data not shown). This suggests that other targets of the apoptotic cascade could be modulated by polyphenol-rich plant extracts. For example, resveratrol was reported to activate SIRT-1, which in turn can modulate proteins involved in cell survival and cell cycle arrest [60]. The exact mechanism by which polyphenol-rich extracts of *A. borbonica* and *D. apetalum* inhibit endothelial cell apoptosis requires further investigation; firstly, to identify the molecules responsible for this protective effect, and secondly, to unveil the involved intracellular signaling pathway.

## 5. Conclusions

Our study shows for the first time that polyphenol-rich extracts from two original medicinal plants from Reunion Island exhibit potent antioxidant and antiapoptotic effects in response to oxLDLs on vascular endothelial cells. The potential use of these plants for prevention of atherosclerosis should be investigated in appropriate preclinical models.

## Figures and Tables

**Table 1:** Global description of medicinal plants tested.

Botanical Name	Family	Voucher Number	Parts Used
*Doratoxylon apetalum ^a^*	Sapindaceae	RUN-055E	Leaf
(Poir.) Radlk			
*Antirhea borbonica ^b^*	Rubiaceae	RUN-052F	Leaf, stem
J.F Gmelin			

Common names: *^a^*: Bois de gaulette; *^b^*: Bois d’Osto.

**Table 2:** Polyphenols identified in *D. apetalum* plant extract.

Peak Number	RT (min)	Compound	Molecular Formula	Mass Error (ppm)	[M-H]^−^	MS/MS Fragments
1	2.1	Protocatechuic acid	C_7_H_6_O4_5_	0	153.0182	109.0282
2	2.3	Gallic acid 4-*O*-glucoside	C_13_H_15_O_10_	2.4	331.0668	168.0053, 125.0231, 211.0238
3	2.7	Unknown	C_15_H_18_O_8_	2.6	325.0927	
4	3.1	Unknown	C_13_H_12_O_8_	2.5	295.0456	163.0389, 112.9867
5	3.3	Procyanidin dimer type B	C_30_H_26_O_12_	5.1	577.1371	289.0714, 407.0766, 125.0231, 109.0282
6	3.7	Epicatechin	C_15_H_15_O_6_	2.7	289.0715	245.0814, 179.0340, 125.0232, 109.0282
7	3.9	Procyanidin trimer type C	C_45_H_38_O_18_	1.2	865.1985	289.0714, 411.0718, 125.0232, 109.0283, 560.0907
8	4.4	Coumaric acid	C_9_H_8_O_3_	0	163.039	119.049
9	4.7	Quercetin 3-*O*-rutinoside (Rutin)	C_27_H_30_O_16_	1.7	609.1461	301.0348
10	4.7	Unknown	C_13_H_12_O_7_	2.7	279.0507	133.0130, 163.0390
11	4.9	Kaempferol 3-*O*-rutinoside	C_27_H_30_O_19_	1.3	593.1509	285.04
12	4.9	Quercetin 3-*O*-hexoside	C_21_H_19_O_12_	0.5	468.0881	301.0347
13	5.1	Kaempferol 3-*O*-hexoside	C_21_H_20_O_11_	2	447.0931	285.0402
14	5.5	Apigenin-7-*O*-rutinoside (Isorhoifolin)	C_27_H_30_O_14_	1.8	577.1563	269.0453
15	5.7	Apigenin hexoside	C_21_H_20_O_10_	1.6	431.0984	269.0453
16	5.9	Kaempferol 3-*O*-(6-malonyl-hexoside)	C_24_H_22_O_14_	1.5	533.0934	489.1035, 285.0401
17	6.5	Apigenin	C_15_H_10_O_5_	3.1	269.0454	

Polyphenol-rich *D. apetalum* extract was analyzed by using a Q Exactive Plus mass spectrometer. Compounds were identified according to their retention time (min)/molecular weight (Da) (see spectra on Figure S1).

**Table 3:** Polyphenols identified in *A. borbonica* plant extract.

Peak Number	RT (min)	Compound	Molecular Formula	Mass Error (ppm)	[M-H]^−^	MS/MS Fragments
1	0.6	Quinic acid	C_6_H_12_O_7_	−0.3	195.0499	127.0388, 111.0438
2	2.1	Protocatechuic acid	C_7_H_6_O_4_	−0.2	153.018	109.0281
3	2.4	5-Caffeoylquinic acid	C_16_H_18_O_10_	0.23	353.0869	191.0549, 179.0337, 173.0442, 135.0437
4	2.6	hydroxybenzoic acid isomer	C_7_H_6_O_3_	−0.29	137.023	93.0331
5	3.3	5-Caffeoylquinic acid	C_16_H_18_O_9_	0.23	353.0869	191.0549, 179.0337, 173.0443, 135.0437
6	3.5	Caffeic acid	C_9_H_8_O_4_	−0.14	179.0337	135.0438
7	4	5-p-Coumaroylquinic acid	C_16_H_17_O_8_	0.2	337.092	191.0549, 173.0442, 93.0331
8	4.1	o/m-Coumaric acid	C_15_H_10_O_4_	−0.2	163.0387	119.0488
9	4.4	p-Coumaric acid	C_15_H_10_O_4_	−0.2	163.0387	119,30,488
10	4.4	5-Feruloylquinic acid	C_17_H_20_O_9_	0.2	367.1026	
11	4.8	Quercetin 3-*O*-rutinoside	C_27_H_30_O_16_	0.1	609.1448	301.0341
12	5	Quercetin 3-*O*-hexoside	C_21_H_20_O_12_	0.1	463.087	300.0268
13	5.1	Kaempferol 3-*O*-galactoside 7-*O*-rhamnoside	C_27_H_30_O_15_	0.1	593.15	284.032
14	5.5	3,5-Dicaffeoylquinic acid	C_25_H_24_O_12_	−0.2	515.1182	191.0550, 179.0338, 353.0870, 135.0438
15	6	Unknown	C_26_H_32_O_14_	−0.01	567.1719	163.0387, 195.0650, 315.1231, 359.1124, 521.1653
16	6	hydroxybenzoic acid isomer	C_7_H_6_O_3_	−0.29	137.023	93.0331
17	6.1	3,4-Dicaffeoylquinic acid	C_25_H_24_O_12_	−0.2	515.1182	353.0870, 173.0443, 191.0549, 135.0437
18	6.2	5-Caffeoylquinic acid	C_16_H_18_O_11_	0.05	353.0868	
19	6.5	1,4/4,5-Dicaffeoylquinic acid	C_25_H_24_O_12_	−0.1	515.1183	173.0443, 353.0869, 191.0549, 135.0436
20	7.3	Quercetin	C_15_H_10_O_7_	0.9	301.0345	
21	8.2	Kaempferol	C_15_H_10_O_6_	1.3	285.0405	

Polyphenol-rich *A. borbonica* extract was analyzed by using a Q Exactive Plus mass spectrometer. Compounds were identified according to their retention time (min)/molecular weight (Da) (see spectra on Figure S2).

**Table 4:** Total polyphenol content of *D. apetalum* and *A. borbonica* plant extracts.

	Total Polyphenol Content (mg GAE/g Plant)
*Doratoxylon apetalum*	3.79 ± 0.14
*Antirhea borbonica*	1.98 ± 0.09

Polyphenols levels were determined by a colorimetric assay and expressed as mg gallic acid equivalent (GAE)/g of dry plant powder. Data are means ± SEM of four independent experiments.

**Table 5:** *D. apetalum* and *A. borbonica* plant extracts prevent lipid peroxidation.

	TBARS (µM)	TBARS (µM)	*p*	TBARS (µM)	*p*	TBARS (µM)	*p*
	control	10 µM		25 µM		50 µM	
*D. apetalum*	4.210	0.046	<0.001	0.023	<0.001	0.027	<0.001
	±0.710	±0.011		±0.012		±0.014	
*A. borbonica*	3.578	0.042	<0.001	0.019	<0.001	0.025	<0.001
	±0.188	±0.003		±0.007		±0.011	
	control	1 µM		5 µM		10 µM	
Curcumin	3.742	3.141	n.s	0.130	<0.001	0.031	<0.001
	±0.572	±0.621		±0.031		±0.011	

## Data Availability

All of the data is contained within the article and the Supplementary Materials.

## Supplementary Materials

The following supporting information can be downloaded at: https://www.mdpi.com/article/10.3390/antiox11010034/s1, Figures S1 and S2: Identification of polyphenols from D. Apetalum and A. Borbonica plant extracts by mass spectroscopy. Figure S3: LDL uptake is not prevented by pre-incubation of *D. apetalum* and *A. Borbonica* in HMEC-1 cells.
