# Supplementary material for: Antioxidant and Cytoprotective Properties of Polyphenol-Rich Extracts from Antirhea borbonica and Doratoxylon apetalum against Atherogenic Lipids in Human Endothelial Cells
Source: Antioxidants (Basel). 2021 Dec 24;11(1):34. doi: 10.3390/antiox11010034 (PMC8773103; doi:10.3390/antiox11010034)

Supplementary materials:

Figure S1: Identification of polyphenols from *D. Apetalum* plant extracts. The polyphenol-rich plant extract was analysed by Q Exactive Plus Orbitrap LC-MS/MS System (315 nm). Compounds were identified according to their retention time (min)/molecular weight (Da).

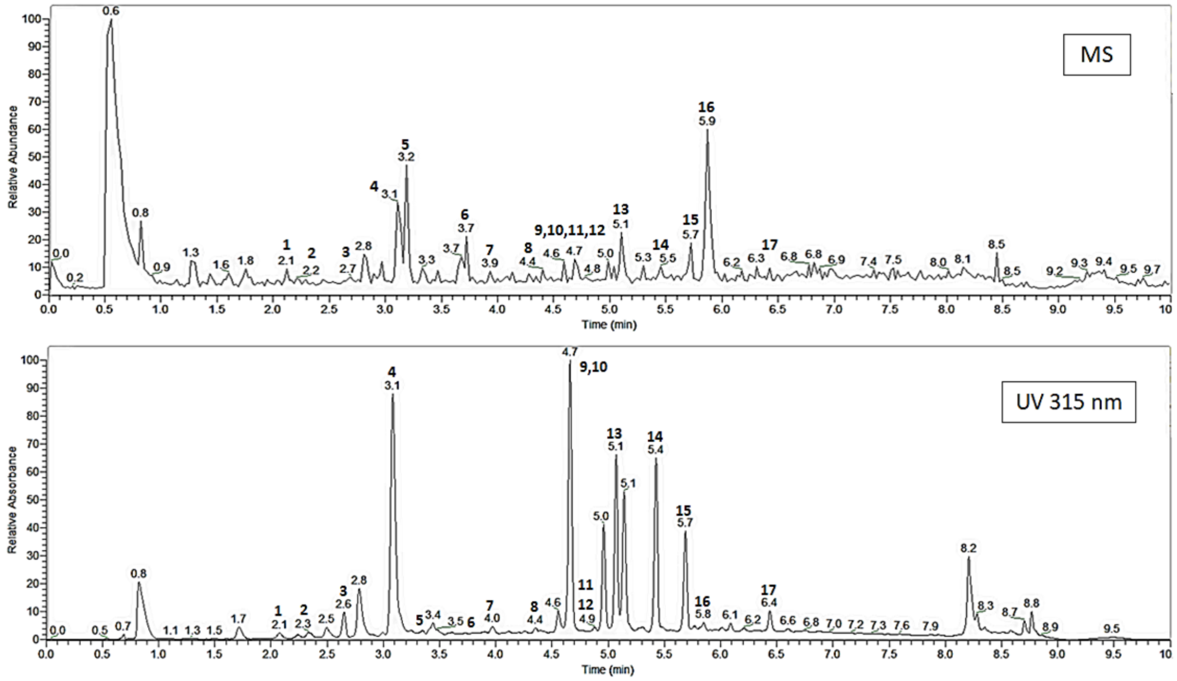

Figure S2: Identification of polyphenols from *A. Borbonica* plant extracts. The polyphenol-rich plant extract was analysed by Q Exactive Plus Orbitrap LC-MS/MS System (315 nm). Compounds were identified according to their retention time (min)/molecular weight (Da).

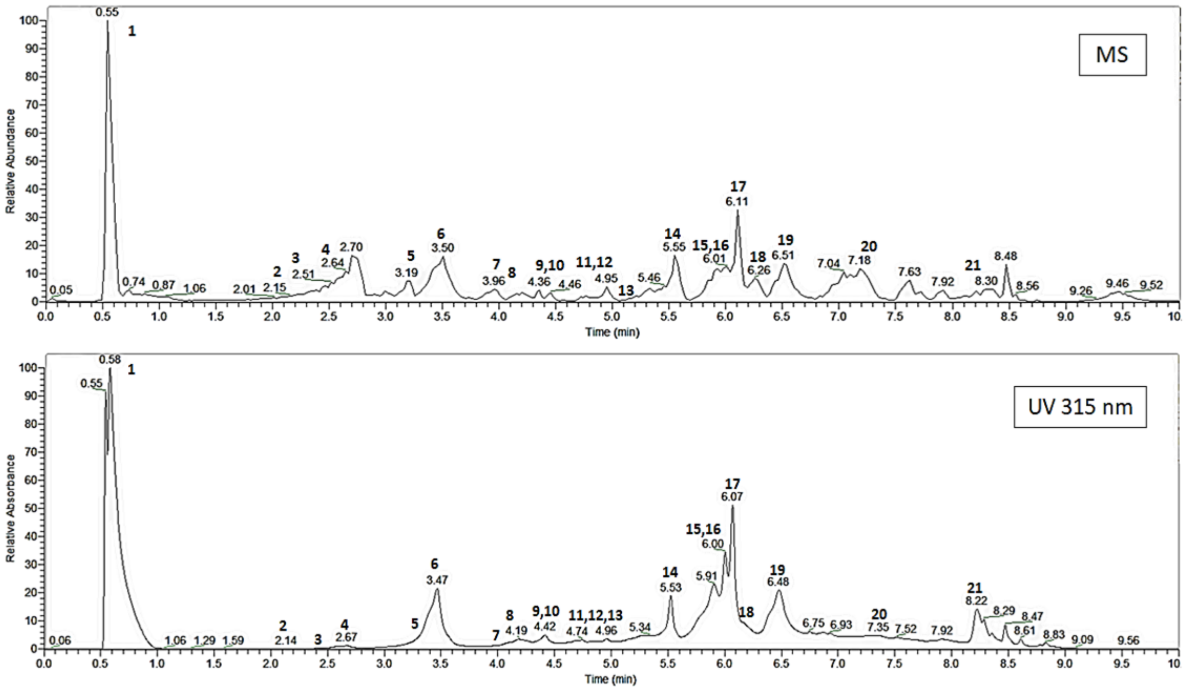

Figure S2: LDL uptake is not prevented by pre-incubation of *D. apetalum* and *A. Borbonica* in HMEC-1 cells.

Uptake of LDL following polyphenol-rich extracts or curcumin pre-treatment. Serum-deprived HMCEC-1 were pre-incubated with polyphenol-rich extracts from *D. apetalum* (25 µM) and *A. borbonica* (25 µM) or curcumin (10 µM) for 2 h before addition of 3,3′-dioctadecyl-indocarbocyanine (DiI)-labelled LDL (30 µL of DiI (10 mg/mL) mixed with 1 mL of LDL (2 mg/mL of apoB); A) Fluorescence of carbocyanine-labelled LDL uptake in HMEC-1 cells (red). Scale bar = 40 µm. After 8h, DiI fluorescence was measured by spectrofluorometry. B) The graph represents the amount of LDL internalized express in arbitrary units (a.u.). Results represent mean ± SEM of three independent experiments. n.s.: no significant difference *vs* control (LDL) using one-way ANOVA with Tukey's post hoc test.

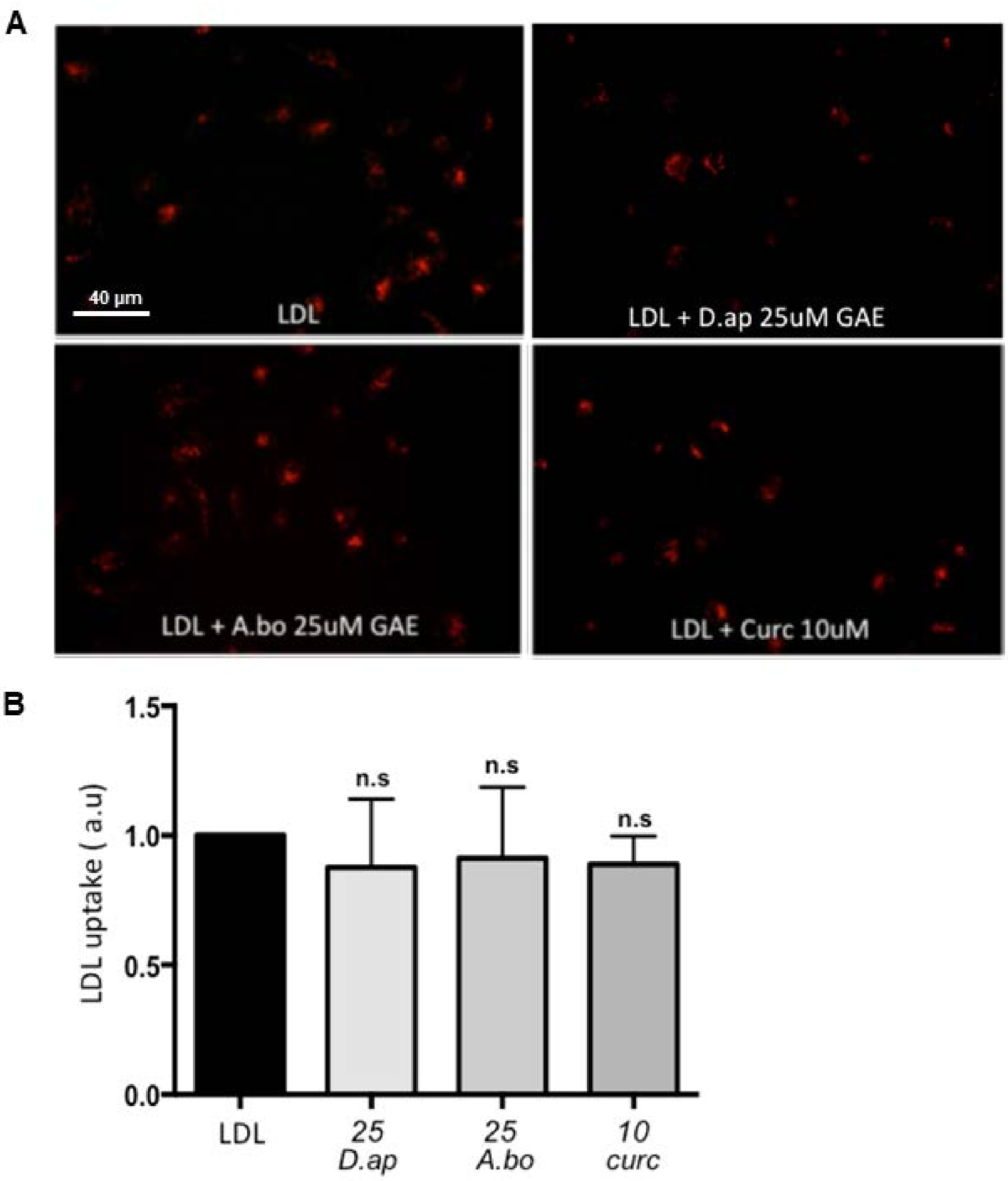

Supplement: Supplementary file 1 [file antioxidants-11-00034-s001.zip › antioxidants-1508179-supplementary.pdf]
